# Study to Identify and Evaluate Predictor Factors for Primary Open-Angle Glaucoma in Tertiary Prophylactic Actions

**DOI:** 10.3390/jpm12091384

**Published:** 2022-08-26

**Authors:** Gabriel Zeno Munteanu, Zeno Virgiliu Ioan Munteanu, Cristian Marius Daina, Lucia Georgeta Daina, Mihaela Cristina Coroi, Carmen Domnariu, Dana Badau, George Roiu

**Affiliations:** 1 Faculty of Medicine and Pharmacy, University of Oradea, 410087 Oradea, Romania; 2 Faculty of Medicine, Lucian Blaga University, 550169 Sibiu, Romania; 3 Petru Maior Faculty of Sciences and Letters, George Emil Palade University of Medicine, Pharmacy, Sciences and Technology, 540142 Targu Mures, Romania; 4 Interdisciplinary Doctoral School, Transilvania University, 500068 Brasov, Romania

**Keywords:** POAG (primary open-angle glaucoma), IOP (intraocular pressure), secondary prevention, risk factor, visual field, visual symptoms, binary logistics regression

## Abstract

The aim of this study is to develop a predictive model with several explanatory variables that can guide ophthalmologists to make a more objective assessment of the evolution of open-angle glaucoma (OAG) during tertiary prevention. Objectives: The evaluation of risk factors and different predictors of symptom progression between patients with POAG and non-glaucoma patients (NG), as well as between primary open-angle glaucoma with high intraocular pressure (POAG) and primary open-angle glaucoma with normal intraocular pressure (NTG), in tertiary prophylactic activities. Methods: This research is an analytical epidemiological study of a prospective cohort. For the study, we took into account personal medical history, physical ophthalmological examination, intraocular pressure (IOP) values, and visual field (VF) parameters, examined with the Opto AP-300 Automated Perimeter using the “fast threshold” strategy. The results of gonioscopy were inconsistently recorded; they were not considered in the study due to missing values, the processing of which would have seriously distorted the statistical analysis. Ophthalmological examination was completed with a dichotomous questionnaire entitled “Symptom Inventory”, made according to the accusations of patients resulting from a “focus group” study. The study was carried out in the ophthalmology office within the Integrated Outpatient Clinic of the Emergency Clinical Hospital of Oradea, Bihor County (IOCECHO) between January–December 2021. The threshold of statistical significance was defined for *p* value < 0.05. The obtained results were statistically processed with specialized software SPSS 22. Results: The study included 110 people, of which 71 (64.54%) had POAG (IOP > 21 mmHg) and 39 people (35.46%) had NTG (IOP < 21 mmHg), the two groups being statistically significantly different (χ^2^ = 9.309, *df* = 1, *p* = 0.002). For the POAG group, glaucomatous loss was early, AD < −6 dB, according to the staging of glaucomatous disease, HODAPP classification. In addition, the groups of POAG and NTG patients was compared with a group of 110 NG patients, these three groups being statistically significantly different (χ^2^ = 34.482, *df* = 2, *p* = 0.000). Analysis of confounding factors (age, sex, residence, marital status) shows a statistically significant relationship only for age (F = 2.381, *df* = 40, *p* = 0.000). Sex ratio for the study groups = 5.11 for OAG and =5.87 for NG. After treatment (prostaglandin analogues and neuroprotective drugs) IOP decreased statistically significantly for both POAG and NTG. Conclusions: this study identified possible predictors of OAG, at the 5% level (risk factors and symptoms as independent variables) using a dichotomous questionnaire tool with a complementary role in tertiary prophylactic activities. The implementation of the focus group interview results as a socio-human research technique will be supportive to clinicians.

## 1. Introduction

Glaucoma is a chronic degenerative disease of multifactorial etiology, characterized by the progressive destruction of the structures of the optic nerve. Clinically, it is manifested by a characteristic, progressive narrowing of the VF with the appearance of blindness in the advanced stages [1,2,3,4,5]. Glaucoma constitutes the first cause of irreversible (permanent) blindness [6,7,8].

The disease presents itself clinically in several forms: POAG is the most frequent manifestation (more than 90% of cases); NTG is a particular form of open-angle glaucoma; secondary open-angle glaucoma (SOAG) is due to other conditions: eye diseases and post-traumatic and iatrogenic conditions [9].

The prevalence of POAG is high; the estimate for the year 2040 states that 112 million people will have glaucoma, and the rate of blindness will be equal between POAG and PACG (primary angle-closure glaucoma) [10]. Prevalence of glaucoma is influenced by race: POAG is more common in black populations and PACG is more prevalent in East Asian populations. Blindness is common in PACG [2]. The 15-year risk of blindness in treated unilateral POAG is 15%, and is 6% in treated bilateral POAG [10].

Early diagnosis and specialized treatment reduce the rate of blindness in glaucoma [11,12].

NTG is the most common subtype of OAG, with possible multifactorial etiology, with IOP statistically considered normal (≤21 mmHg) [13,14,15,16].

By extrapolating data from the European level, the official statistics from Romania estimate the number of glaucoma patients at 140,000 people, of which 132,000 are diagnosed with POAG [5]. The only effective treatment in glaucoma is lowering the IOP to preserve visual function.

Prevention in public health involves levels of intervention (types of prevention) [17,18]. For glaucoma, secondary prevention is represented by early diagnosis to avoid the unfavorable course of the disease [3,19]. Tertiary prevention acts to prevent and reduce complications after the onset of the disease, to reduce injuries and inflammations, prevent relapses and suffering, and to adapt the patient to the incurable situation through adequate treatment [18].

The sociological tool, the dichotomous questionnaire “Symptom Inventory” was made following a “focus group” study. A focus group is a technique of qualitative sociological research, which derives from “focused interviews” or “in-depth group interviews” and is defined as a group of interacting individuals who have some common interests or characteristics, gathered by a moderator, who uses it to obtain information about a specific issue. In another approach, it is considered an informal discussion group between selected people on a certain topic. The method has an exploratory character highlighted by identification of problems, perceptions, opinions, reactions, behaviors, and motivations in a real situation. It is a group discussion attended by 6 to 10 people [20,21,22].

This technique is used in connection with other methods, especially with questionnaire-based surveys and individual interviews, as a way of combining methods. A focus group is first conducted to identify issues and questions that will then be included in a questionnaire. The focus group is used as the main method, and the survey becomes a helpful method that verifies the relevance of the issues established by the researcher for the group discussions. Thus, the advantages of using a focus group include real-life data in a concrete environment and it being a flexible technique with a high validity that produces results quite quickly and has low costs.

The disadvantages could be the following: it provides the researcher with less control (compared to an individual interview, for example); sometimes the data are difficult to analyze; it requires special skills and knowledge from the researcher; differences between groups can be distorted, organizing groups can be quite difficult, and discussions should be conducted in such a way as to encourage interaction between group members [23,24]. The aim of the study is to develop a predictive model with several explanatory variables that can guide ophthalmologists to make a more objective assessment of the evolution of open-angle glaucoma (OAG) during tertiary prevention.

Objectives: The evaluation of risk factors and different predictors of symptom progression between patients with POAG and non-glaucoma patients (NG), as well as between primary open-angle glaucoma with high intraocular pressure (POAG) and primary open-angle glaucoma with normal intraocular pressure (NTG), in tertiary prophylactic activities.

## 2. Materials and Methods

### 2.1. Ethical and Legal Issues

In order to carry out the study, the approval of the Ethical Council Opinion (Document no. 8630/03.04.2019) and of the Ethics Commission Opinion (Document no. 8805/04.04.2019) were requested and obtained, as well as unrestricted access to archived patient data for research purposes from the scientific department (FOCG) within the Oradea County Emergency Hospital.

### 2.2. Data Collection

The study took place between January and December 2021 using only the information available from the medical archive of glaucoma patients from the ophthalmology office within the Integrated Outpatient Clinic of the Oradea Emergency Clinical Hospital (IOCECHO), Bihor County.

The results obtained when applying the “Symptom Inventory” questionnaire by the patients were recorded and processed statistically.

### 2.3. Study Design

The present work is an analytical epidemiological study of a prospective cohort [25].

### 2.4. Methodology

The study was carried out at the ophthalmology office of the IOCECHO structure as part of the permanent activities dedicated to the active detection and treatment of glaucoma patients. Every year in March, the activities for the detection of glaucoma patients are promoted in the local press, occasioned by campaigns regarding World Glaucoma Week, supported by the Romanian Glaucoma Society [5]. The medical and statistical data obtained in these secondary prevention activities refer only to the investigated population.

The present study included all glaucoma patients diagnosed, treated, and monitored in this medical unit, as well as healthy people who participated in the organized screening.

The glaucoma patients belonged to the two clinical forms: POAG and NTG patients diagnosed with glaucoma (POAG and NTG) who did not have other eye diseases and did not undergo medical treatment or surgery [6].

Other forms of open-angle glaucoma were excluded: juvenile open-angle glaucoma (JOAG); SOAG: pseudo-exfoliative, pigmented, with crystalline particles, associated with intraocular tumors; uveitic, neovascular, associated with intraocular tumors, retinal detachment, post-traumatic corticosteroid-induced, and surgical and/or laser treatment. Other eye diseases such as corneal, lens, vitreous, and retinal diseases, etc., were excluded.

Epidemiological, demographic, and specialized ophthalmological parameters were used to characterize the health status of patients with OAG. The epidemiological parameters were number of disease cases, number of non-glaucoma people, age, sex, sex ratio, place of residence, marital status, and education level.

The examination of the patients was performed by two methods: by interview and by specialized medical investigation completed with a sociological tool, a dichotomous “Symptom Inventory” questionnaire resulting from a focus group study. The questionnaire included the ten questions (two questions referring to nonvisual symptoms and eight to visual symptoms) most frequently proposed by the focus group participants, patients diagnosed with OAG (POAG and NTG).

Glaucoma patients were referred by ophthalmologists for monitoring and counseling, and depending on adherence, specific treatment was initiated. The data of the considered medical interrogation were family medical history and pathological personal history and of the associated diseases. The objective ocular examination consisted of the determination and recording of IOP with Goldmann aplanotonometer. IOP was considered an important indicator both for the detection of glaucomatous disease and in monitoring its progression under treatment. The ocular functional examination consisted of the determination of visual acuity and the determination of the visual field. Visual acuity was investigated with the Snellen chart. The determination of the visual field was performed with the Opto AP-300—Computerized Perimeter, with the “fast threshold” strategy, using optical correction as needed. The following parameters were considered: credibility indices (“false-positive” answers, “false-negative” answers), time required to perform the test, theoretical “visual slope to 10°”, “zero level”, the structural defect (PD—pattern defect), the average defect (average defect—AD), and the graph of the defect (Bebie curve). For the statistical interpretation of the graph of the centralized defect of a test result (Bebie curve), we used the following categorical classification (Table 1) [26].

The criteria for including patients in the study were credibility indices, with a percentage threshold and additional qualitative descriptions (maximum 15% for “false-positive errors” and “false-negative errors”).

The sociological tool of this study, the dichotomous questionnaire entitled “Symptom Inventory” was compiled following a focus group study that took place between 19 February–15 March 2021.

A “focus group” is an attractive and effective qualitative method of investigation. Leadership of the focus group occurs in three phases:

The conceptualization phase, which requires determining the purpose of the research, collecting data on experiences, beliefs, attitudes, and needs related to certain issues;

- The interview phase, which begins with the elaboration of the questions, and must have a note of spontaneity. The focus group is led and modeled by a researcher with the role of facilitating the discussion without actively participating, and who has knowledge about the problem and who is supported by an assistant (co-leader) with technical duties (organization, reception of participants, registration);
- The phase of analysis and drafting of the report: qualitative analysis of the data must be systematic and verifiable and will process the collected data (transcription, analysis, and comparison will be conducted as a whole, not individually, and between groups, not within a group). Five factors will be taken into account when interpreting an analysis: words, context, internal consistency, specificity of answers, and discovery of important (key) ideas. The report must be descriptive and interpretive, presenting the meaning of the data, not a summary of it.

The elaboration and testing of the interview guide is mandatory in organizing a focus group. The interview guide is a series of logical questions of the funnel type (from general to very specific), with the role of satisfying the established objectives and collecting a sufficiently large volume of information for analysis and obtaining in-depth information related to the studied topic, being constructed like a scenario, following various problems, questions, or situations that the participants have to face. The group must be structured; the moderator follows the issues to be discussed and the interactions between the members of the group. A higher number of problems means a higher degree of structuring [27,28,29,30].

The focus group development involves establishing the topic of discussion and the structure of the group and the ways of selecting the participants; elaboration and testing of the interview guide; determining the date, place, and preparation for the meeting; training the moderator and the assistant moderator; and focusing the group. Different types of questions are used in this technique: opening questions, introductory questions (“warm-up” questions), intermediate questions, key questions, and final questions.

After three meetings, at the end, the main symptoms were identified and the questionnaire was completed and discussed with the participants. Ten main symptoms were identified that made up the “Symptom Inventory” questionnaire, which was then applied to patients with OAG (POAG and NTG) and healthy people [24,31,32,33,34,35,36,37,38,39,40,41,42,43].

### 2.5. Statistical Analysis

We analyzed indicators of central tendency (mean) and dispersion (SD).

The study of the distribution of ordinal variables was carried out with the parametric Kolmogorov–Smirnov and Shapiro–Wilk tests, and with the nonparametric tests, the Wilcoxon test for related scores was used. The Chi-square test was used for categorical variables described as frequencies in testing the equality of two or more proportions.

Confusion is the distortion of the measure of the effect of an exposure defined as risk, due to the exposure with a factor/factors that may influence the development of the studied disease. The estimation of the association with the simultaneous control of several confounding factors (age, sex, domicile, marital status) can be performed by univariate analysis [44]. Regression is a statistical prediction procedure in which we use a variable called a predictor (independent variable) to predict the values of a variable called a criterion (dependent variable) [45]. We used binomial logistic regression in our epidemiological study to identify individual characteristics associated with disease development by creating a prediction model of probabilistic association of criterion values with those of predictors [45].

This binary logistic model allowed us to statistically determine the parameters (questionnaire symptoms) that significantly predicted POAG and NTG. The basic concept in logistic regression is the odds ratio that expresses the probability of an event occurring/not occurring and quantifies the impact of the predictor on the criterion. The *p* value < 0.05 was considered statistically significant. Statistical analysis was performed with the program IBM SPSS Statistics Version 22 [45,46,47].

## 3. Results

The study included 110 people, of which 71 (64.54%) had POAG (IOP > 21 mmHg) and 39 people (35.46%) had NTG (IOP < 21 mmHg), the two groups being statistically significantly different (χ^2^ = 9.309, *df* = 1, *Sig.* = 0.002). For the POAG group, glaucomatous loss was early, AD < −6 dB, according to the staging of glaucomatous disease, HODAPP classification (Hodapp–Parrish–Anderson criteria) [2]. In addition, the group of POAG and NTG patients was compared with a group of 110 NG patients, the three groups being statistically significantly different (χ^2^ = 34.482, *df* = 2, *Sig.* = 0.000). The classification of patients according to the risk of IOP values was: 8 people (11.26%) without risk of IOP < 21 mmHg; 14 people (19.71%) with low IOP risk = 22–23 mmHg, 42 people (59.15%) with moderate IOP risk = 24–29 mmHg; 7 people (9.85%) at high risk of IOP > 30 mmHg. The distribution of demographic indicators of patients is presented in Table 2.

Analysis of confounding factors (age, sex, residence, marital status) performed with univariate analysis shows a statistically significant relationship only for age (F = 2.381, *df* = 40, *Sig.* = 0.000) but not for sex (F = 0.390, *df* = 1, *Sig.* = 0.534), residence (F = 1.287, *df* = 1, *Sig.* = 0.259), and marital status (F = 1.498, *df* = 3, *Sig*. = 0.220) [44]. The sex ratios for the study groups are as follows: for OAG = 5.11, for POAG = 6.88, for NTG = 3.33, and for NG = 5.87. The family medical history of patients with POAG presents hypertension, (first-degree relatives) with a frequency of 10 people (men)—14.08%; and diabetes mellitus Type II (first-degree relatives), with a frequency of 3 people (men)—4.22%.

Previous diseases were hypertension, with a frequency of 4 people—5.63% (4 men); and diabetes mellitus Type II, with a frequency of 3 people—4.22% (3 men); allergy, in 3 people—4.22% (2 men and 1 woman); resting migraine and angina pectoris, in 1 person—1.40% (1 man); hyperlipemia with hypercholesterolemia, in 1 person—1.40% (1 man).

The ophthalmological conditions of glaucoma patients were myopia, corrected in 4 people—5.63% (2 men and 2 women); and corrected hypermetropia, in 6 people—8.45% (4 men and 2 women). From the family medical history recorded in the monitoring sheets of patients with NTG, the following can be retained: hypertension (first-degree relatives), with a frequency of 5 people (3 men and 2 woman)—12.82%; diabetes mellitus Type II (first-degree relatives), with a frequency of 1 person (men)—2.56%; glaucoma (first-degree relatives), in 1 person (men)—2.56%; and blindness (first-degree relatives), in 1 person (men)—2.56%.

Previous diseases were hypertension, with a frequency of 1 person—2.56% (1 woman); diabetes mellitus Type I, with a frequency of 1 person—2.56% (1 woman); diabetes mellitus Type II, with a frequency of 2 people—5.12% (2 women); allergy, in 4 people—10.25% (3 men and 1 woman); headache, in 2 people—5.12% (1 men and 1 woman); and migraine, in 1 person—2.56% (1 woman).

Pre-existing eye conditions were myopia, corrected in 6 people—15.38% (4 men and 2 women); and corrected hypermetropia, in 5 people—12.82% (5 men). To classify patients as individuals in one of the study groups, the arithmetic mean of the IOP between the right eye (RE) and the left eye (LE) was calculated.

All patients received topical ocular hypotensive treatment with prostaglandin analogues in combination with neuroprotective drugs and did not undergo surgical treatment. After treatment, IOP decreased statistically significantly: for POAG, by 3.61 mmHg (14.19%), from 25.44 ± 3.51 mmHg to 21.83 ± 5.26 mmHg (z = −2.763 ^b^; *p* = 0.006); and for NTG, by 1.14 mmHg (6.72%), from 16.94 ± 2.40 mmHg to 15.80 ± 2.68 mmHg (z = −4.151; *p* = 0.000) (Table 3) [2].

Examination of visual acuity (VA) and analysis of VF parameters constituted the ocular functional examination. Optical correction was required for 50 persons (35.21%) with POAG and 16 persons (21.51%) with NTG. The statistical study of the differences between VF parameters obtained at the computerized perimeter between the first and last consultation the patients with POAG, NTG, and NG patients is presented in Table 4 and Table 5.

Using the Bebie curve graph, a rapid assessment of the integrity of the visual field in relation to age was made in patients with POAG, NTG, and NG at the first consultation (Table 6).

Distribution of the Bebie curve graph shows a predominance of the type III model for both eyes. The Chi-square test showed a statistically significant difference between the types of Bebie curve indicators for RE and LE. In POAG: RE—χ^2^ = 167.704, *df* = 3, *Sig.* = 0.000; LE—χ^2^ = 147.085, *df* = 3, *Sig.* = 0.000). In those with NTG: RE—χ = 16.026, *df* = 1, *Sig.* = 0.000; LE—χ^2^ = 38.000, *df* = 2, *Sig.* = 0.000). For the study of predictive factors for POAG and NTG, we developed a binary logistics model to determine which analysis parameters were identified as significant predictor risk factors for POAG (Table 7, Table 8, Table 9 and Table 10).

Variable IOP is a risk factor for POAG, being a significant predictor at 5%. The probability of a person falling into the POAG category increases by 41.79%. If we stratify the variable “age” by nodal age groups at five-year intervals from 40 to 60 years, we obtain high OR values from Exp (B) = 0.284, 95% CI = 0.158–0.507, up to “age over 55 years old” Exp (B) = 0.109, 95% CI = 0.157–0.210; of statistical significance (*Sig.* = <0.05). Age, especially the nodal value of 55 years, is a risk factor for POAG, being a significant predictor at the level of 5% (Exp (B) = 0.284, 95% CI = 0.158–0.507, *Sig.* = <0.05).

The final regression model states that the risk factors with a significant predictor role at the level of 5% (*Sig.* = <0.05) are IOP, which is the most important risk factor; age “over 55 years”; and VF indicators (“false-negative” errors, “slope at 10°”, “zero level”, and PD).

The final regression binary logistics model states that the following risk factors are significant predictors at the 5% level: IOP for POAG; “age over 55 years” for POAG; and NTG and VF indicators (“false negative”, “slope at 10°”, “zero level”, and PD for POAG; and “false negative”, “zero level”, and PD for NTG). 

Questionnaire Assessment: “Symptom Inventory” shows the comparative distribution of affirmative responses (certifying the presence of the symptom) within POAG and NTG patients (Table 11). The variables included in the symptom questionnaire were considered predictors for a statistical model that specifies the individual characteristics associated with morbid conditions (Table 12, Table 13, Table 14 and Table 15).

The probability that a patient with OAG will be included in the POAG group based on the binomial logistic regression model (symptom = sensation of tension in the eye/eye strain) is 16.08%. The accuracy of the classification for IOP was 84.5% for POAG and 28.2% for NG patients, with an overall accuracy of 56.4%.

Assessing the differences between POAG (POAG and NTG) and NG considering the values of Exp (B), the final regression model states that “sensation of dry eyes”, “sensation of tension in the eye/eye strain”, “difficulty in short-distance sight”, “difficulty in remote view (to see at a distance)”, “ebluisare—blindness in bright light” and “ebluisare—blindness passing from light to darkness” are significant predictors at 5%.

The differences between POAG and NG on the one hand and NTG and NG on the other hand show only the additional presence in POAG of the symptoms “difficulty in remote view (to see at a distance)” and “ebluisare—blindness in bright light”. The differences between the two clinical forms of glaucomatous disease (POAG and NTG) are only in the “sensation of tension in the eye/eye strain” symptom (Exp (B) = 0.202, 95% CI = 0.044–0.934, *Sig.* = <0.05).

The final regression model for “Symptom Inventory” states that the following independent variables are significant predictors for POAG at the 5% level: “Sensation of dry eyes”(nonvisual symptom), “sensation of tension in the eye/eye strain”, “difficulty in short-distance sight”, “difficulty in remote view (to see at a distance)”, “ebluisare—blindness in bright light” and “ebluisare—blindness passing from light to darkness”. The probability of a person falling into the OAG (POAG + NTG) category increases for “ebluisare—blindness in bright light” by 32.65%, “sensation of tension in the eye/eye strain” by 31.78%; “difficulty in short-distance sight” by 30.60%; “sensation of dry eyes” by 29.02; “ebluisare—blindness passing from light to darkness” by 27.06%, and “difficulty in remote view (to see at a distance)” by 21.50%.

The average age of the studied groups was between 40.72 ± 6.62 and 48.59 ± 5.33, which are the ages at which refractive issues necessitate adequate optical correction. Despite the fact that not all patients mentioned difficulties with distance vision in the questionnaire, statistical analysis of the VA test results shows that 26 patients (23.63%) of POAG require optical correction (18 POAG patients—25.35% and 8 NTG patients—20.51%). Tearing is the dominant nonvisual symptom, present in 49 people (44.55%) with POAG. For the visual symptoms in OAG patients, the positive response to the symptom “sensation of intraocular pressure” achieves the largest difference between the two groups: 15 people (21.13%) with POAG compared to 2 people (5.13%) with NTG. The statistical model of binomial logistic regression allowed for the consideration of variables from the symptom questionnaire as predictors specifying individual characteristics associated with morbid conditions.

## 4. Discussion

Several risk factors and predictors for POAG have been reported in the literature, and the most important factors are advanced age and high IOP [48]. In POAG, IOP remains the main risk and the most consistent risk factor for glaucoma assessment and progression; age and familiarity are also great risk factors. IOP is the only factor that can be modified, being a modifiable risk in order to treat the disease, either medically or surgically. For each single mmHg increase it has been consistently attributed a 10% higher risk [49,50,51]. The baseline risk factors could help in identifying those at highest risk of POAG incidence [52,53]. Both increasing age and greater IOP increase the odds of VF progression by 30% (for each 5-year increment in age and 1 mmHg increase in IOP fluctuation) [54]. It has also been observed in glaucoma patients that high false-negative rates are statistically significantly associated with progression of VF parameters, without being influenced by age, race, sex, or socioeconomic status [55]. The development of techniques and protocols for the investigation of VF may increase the accuracy of the detection of disease progression and therapeutic conduct and improve quality of life [56].

Predictive statistical models are useful in the development of the study of glaucomatous disease. The development of predictive models uses one or more explanatory variables. The need for predictive models can help clinical ophthalmologists to make a more objective assessment of risk [57]. Glaucoma blindness was due to late diagnosis and disease progression, although target IOP (high baseline AD and IOP and advanced age) was maintained [58].

The population must be informed about the natural evolution of the disease and the effects of the treatment; it must be referred to specialized medical assistance to monitor functional and structural changes [59]. Population-based glaucoma screening activities need to develop innovative approaches with strategies adapted to target groups [60].

The current variant of the strategy proposed following this research for tertiary prophylactic actions for POAG is the use of a complementary method of such a dichotomous questionnaire containing an inventory of specific symptoms for identification of possible predictors that can help clinical ophthalmologists to make a more objective assessment of risk.

A similar study with the dichotomous “Symptom Inventory” questionnaire of specific symptoms was performed to detect ocular hypertension during secondary prophylactic activity (HTO). The differences found by using “Symptom Inventory” for OHT and OAP showed the presence of three common symptoms: “Sensation of intraocular pressure”, “sensation of dry eyes” and “difficulty in short-distance sight”. “Sensation of intraocular pressure” was a significant predictor at the 5%, for OHT (Exp (B) = 0.093, 95% CI = 0.014–0.603, *Sig.* = <0.013) and for POAG (Exp (B) = 0.466, 95% CI = 0.240–0.904, *Sig*. = <0.024) [61]. The advantage of using the “Symptom Inventory” questionnaire lies in three essential elements: it is cheap, easy to apply, and surprises evolution in dynamics.

The current complementary procedure proposed involves completing the questionnaire in about a maximum of 1–2 min (at the ophthalmologist, family doctor, or the occupational physician, during a consultation, or by mail, telephone, or through social media) and depending on the result, guiding the patient to a specialized medical service. The “Symptom Inventory” questionnaire can be improved by further additional extensive research. In Romania, the phenomenon of population aging causes an increase in morbidity and mortality in the context of the increase in the prevalence of chronic diseases under the influence of health determinants [62,63,64,65,66].

The limitations of the procedure described in this paper are the difficulty for the questionnaire in detecting the change in specific symptoms that are discrete in type, changes often not taken into account by the patient, the patient’s willingness to communicate with the doctor, and reluctance to results obtained by sociometric methods.

## 5. Conclusions

This study identified possible predictors of OAG at the 5% level (risk factors and symptoms as independent variables) using a dichotomous questionnaire tool with a complementary role in tertiary prophylactic activities. The implementation of the focus group interview results as a socio-human research technique will be supportive to clinicians.

## Figures and Tables

**Table 1:** Classification of the centralized defect of a VF test result (Bebie curve).

Bebie curve type I	Extensive and deep damage to the visual field
Bebie curve type II	No real defects in the visual field
Bebie curve type III	Small but deep defects in the visual field
Bebie curve type IV	A visual field with a very large and shallow defect

VF—visual field.

**Table 2:** Distribution of demographic indicators of patients.

Parameters		Results					
		Primary Open-Angle Glaucoma Patients		Primary Open-Angle Glaucoma with Normal Intraocular Pressure Patients		Non-Glaucomatous Subjects	
		*n*	%	*n*	%	*n*	%
Number of cases		71	100	39	100	110	100
Sex	Male	62	87.32	30	76.93	94	85.45
	Female	9	12.68	9	23.07	16	14.55
Age		44.76 ± 7.62	Min = 35 Max = 62	40.72 ± 6.62	Min = 35 Max = 59	48.59 ± 5.33	Min = 35 Max = 61
Residence	Urban area	48	67.60	24	61.53	74	67.27
	Rural area	23	32.40	15	38.47	36	32.73
Marital status	Married	44	61.97	19	48.72	70	63.64
	Unmarried	18	25.35	17	43.58	17	15.45
	Widowed	2	2.82	0	0.00	3	2.73
	Divorced	7	9.86	3	7.70	20	18.18
Studies	Primary cycle	5	7.04	2	5.13	6	5.45
	Gymnasium cycle	3	4.23	3	7.69	8	7.27
	Professional school	8	11.27	2	5.13	7	6.36
	High school	29	40.85	17	43.59	50	45.45
	Post-high school	3	4.23	2	5.13	6	5.45
	Higher education	18	25.35	9	23.08	25	22.73
	Post-university	5	7.04	4	10.26	8	7.27

*n*—number, %—percent, Min—minimum, Max—maximum.

**Table 3:** Distribution of IOP parameters in POAG and NTG patients, at the first and last consultation.

Parameters	Initial Consultation	Final Consultation	z	*p* *
POAG-IOP-(BE)	25.44 ± 3.51	21.83 ± 5.26	−2.763 ^b^	0.006
NTG-IOP-(BE)	16.94 ± 2.40	16.10 ± 2.55	−3.141 ^b^	0.002

^b^—based on positive ranks, BE—both eyes = (RE + LE); * Wilcoxon Test.

**Table 4:** Distribution of credibility indices in the interpretation of the visual field result between the first and last consultation in POAG, NTG, and NG patients.

Indicators	Initial Consultation	Final Consultation	z	*p* *	Considered Values
Average duration (minutes)—POAG	10.00 ± 2.50	10.86 ± 2.14	−4.292 ^b^	0.000	
Average duration (minutes)—NTG	9.61 ± 2.62	10.90 ± 2.10	−5.033 ^b^	0.000	
Average duration (minutes)—NG	6.82 ± 1.51				
False positive—POAG(BE)	3.90 ± 5.31	5.93 ± 6.11	−3.221 ^b^	0.000	≤15%
False positive—NTG (BE)	3.33 ± 5.08	5.51 ± 6.11	−3.187 ^b^	0.001	≤15%
False positive—NG (BE)	3.14 ± 5.28				≤15% (≤10)%
False negative—POAG (BE)	5.33 ± 6.05	5.61 ± 6.33	−1.370 ^b^	0.171	≤15%
False negative—NTG (BE)	6.17 ± 7.32	6.04 ± 6.59	−1.946 ^b^	0.052	≤15%
False negative—NG (BE)	3.45 ± 5.50				≤15% (≤10)%

^b^—based on positive ranks, BE—both eyes = (RE + LE); * Wilcoxon Test; z—two-related-samples Wilcoxon test; *p*—level of statistical probability.

**Table 5:** Distribution of VF parameters result between the first and last consultation in POAG, NTG, and NG patients.

Parameter	Initial Consultation	Final Consultation	z	*p* *
Tested points—POAG (BE)	372.65 ± 109.75	370.80 ± 97.97	−1.070 ^b^	0.285
Tested points—NTG (BE)	348.60 ± 111.11	381.30 ± 110.79	−3.196 ^b^	0.001
Tested points—NG (BE)	293.44 ± 41.20			
Visual slope at 10°—POAG (BE)	2.24 ± 0.99	1.86 ± 0.81	−5.713 ^b^	0.000
Visual slope at 10°—NTG (BE)	2.43 ± 1.10	1.84 ± 0.92	−4.675 ^b^	0.000
Visual slope at 10°—NG (BE)	2.68 ± 0.72			
Zero Level—POAG (BE)	22.31 ± 5.46	24.58 ± 5.53	−6.098 ^b^	0.000
Zero Level—NTG (BE)	21.85 ± 5.76	23.87 ± 5.86	−4.223 ^b^	0.000
Zero Level—NG (BE)	24.97 ± 2.60			
PD—POAG (BE)	2.45 ± 2.92	2.48 ± 1.80	−2.625 ^b^	0.009
PD—NTG (BE)	2.19 ± 2.60	2.73 ± 2.58	−3.765 ^b^	0.000
PD—NG (BE)	0.29 ± 0.38			
AD—POAG (BE)	−0.51 ± 3.92	0.52 ± 4.11	−4.463 ^b^	0.000
AD—NTG (BE)	−0.06 ± 3.48	0.21 ± 4.34	−0.445 ^b^	0.656
AD—NG (BE)	−0.02 ± 0.13			

BE—both eyes = (RE + LE); z—two-related-samples Wilcoxon test; *p*—level of statistical probability; ^b^—based on negative ranks; * Wilcoxon signed ranks test.

**Table 6:** Distribution of Bebie curve from the visual field examination for POAG and NTG patients at the first consultation.

Bebie Curve Modes	Primary Open-Angle Glaucoma Patients				Primary Open-Angle Glaucoma with Normal Intraocular Pressure Patients			
	RE		LE		RE		LE	
	*n*	%	*n*	%	*n*	%	*n*	%
Bebie curve type I	2	2.82	3	4.23	2	5.13	0	0.00
Bebie curve type II	2	2.82	3	4.23	0	0.00	0	0.00
Bebie curve type III	65	91.54	62	87.31	31	79.49	32	82.05
Bebie curve type IV	2	2.82	3	4.23	6	15.38	7	17.95
Total	71	100	71	100	39	100	39	100

*n*—number of cases; %—percent.

**Table 7:** Distribution of binomial logistic analysis results for OAG (POAG and NTG) and NG patients at the first consultation.

Risk Factor	Parameter Estimate	SE	Wald χ^2^	*df*	*Sig.*	Exp (B)	95% CI	
							Lower	Upper
IOP (arithmetic mean for POAG, NTG, NG)	−0.331	0.046	52.722	1	0.000	0.718	0.657	0.785
Age	0.111	0.019	35.087	1	0.000	1.117	1.077	1.159
Age (>40 years/<40 years)	−1.743	0.386	20.350	1	0.000	0.175	0.082	0.373
Age (>45 years/<45 years)	−2.214	0.334	44.041	1	0.000	0.109	0.057	0.210
Age (>50 years/<50 years)	−1.260	0.297	18.016	1	0.000	0.284	0.158	0.507
Age (>55 years/<55 years)	−1.518	0.376	16.251	1	0.000	0.219	0.105	0.458
Age (>60 years/<60 years)	−2.070	0.635	10.613	1	0.001	0.126	0.036	0.438
Duration of VF performing	0.034	0.098	0.119	1	0.730	1.034	0.854	1.252
False positive	−0.044	0.031	2.089	1	0.148	0.957	0.901	1.016
False negative	−0.115	0.027	18.009	1	0.000	0.891	0.845	0.940
Tested points	−0.003	0.003	0.889	1	0.346	0.997	0.991	1.003
Slope 10°	−0.409	0.199	4.240	1	0.039	0.664	0.450	0.981
HOV-Zero level	0.181	0.058	9.768	1	0.002	1.198	1.070	1.342
PD	−1.670	0.416	16.138	1	0.000	0.188	0.083	0.425
AD	1.004	0.611	2.703	1	0.100	2.729	0.825	9.028

**Table 8:** Distribution of binomial logistic analysis results for POAG and NG patients at the first consultation.

Risk Factor	Parameter Estimate	SE	Wald χ^2^	*df*	*Sig.*	Exp (B)	95% CI	
							Lower	Upper
IOP (median POAG, NG)	−1.534	0.375	16.742	1	0.000	0.216	0.103	0.450
Age	0.091	0.020	20.547	1	0.000	1.096	1.053	1.140
Age (>40 years/<40 years)	−1.296	0.426	9.233	1	0.002	0.274	0.119	0.631
Age (>45 years/<45 years)	−1.912	0.360	28.167	1	0.000	0.148	0.073	0.299
Age (>50 years/<50 years)	−0.972	0.326	8.923	1	0.003	0.378	0.200	0.716
Age (>55 years/<55 years)	−1.250	0.410	9.308	1	0.002	0.286	0.128	0.639
Age (>60 years/<60 years)	−1.617	0.640	6.389	1	0.011	0.199	0.057	0.695
Duration of VF performing	0.117	0.116	1.021	1	0.312	1.124	0.896	1.411
False positive	−0.056	0.034	2.770	1	0.096	0.945	0.884	1.010
False negative	−0.108	0.030	13.157	1	0.000	0.898	0.847	0.052
Tested points	−0.003	0.004	0.857	1	0.355	0.997	0.990	1.004
Slope 10°	−0.432	0.236	3.358	1	0.067	0.649	0.409	1.030
HOV-zero level	0.142	0.065	4.803	1	0.028	1.153	1.015	1.310
PD	−1.035	0.420	6.075	1	0.014	0.355	0.156	0.809
AD	3.082	1.229	6.288	1	0.012	21.803	1.960	242.511

**Table 9:** Distribution of binomial logistic analysis results for NTG and NG patients at the first consultation.

Risk Factor	Parameter Estimate	SE	Wald χ^2^	*df*	*Sig.*	Exp (B)	95% CI	
							Lower	Upper
IOP (median NTG. MG)	−0.041	0.062	0.425	1	0.514	0.960	0.850	1.085
Age (>40 years/<40 years)	−2.457	0.462	28.313	1	0.000	0.086	0.035	0.212
Age (>45 years/<45 years)	−2.835	0.456	38.722	1	0.000	0.059	0.024	0.143
Age (>50 years/<50 years)	−1.953	0.516	14.355	1	0.000	0.142	0.052	0.390
Age (>55 years/<55 years)	−2.238	0.753	8.824	1	0.003	0.107	0.024	0.467
Duration of VF performing	−0.089	0.124	0.518	1	0.472	0.914	0.717	1.167
False positive	−0.019	0.049	0.161	1	0.688	0.981	0.892	1.079
False negative	−0.149	0.038	15.181	1	0.000	0.861	0.799	0.028
Tested points	−0.002	0.005	0.297	1	0.586	0.998	0.989	1.006
Slope 10°	−0.459	0.276	2.773	1	0.096	0.632	0.368	1.085
HOV-zero level	0.253	0.080	9.903	1	0.002	1.288	1.100	1.507
PD	−2.524	0.520	23.552	1	0.000	0.080	0.029	0.222
AD	0.859	0.695	1.528	1	0.216	2.360	0.605	9.214

**Table 10:** Distribution of binomial logistic analysis results for POAG and NTG patients at the first consultation.

Risk Factor	Parameter Estimate	SE	Wald χ^2^	*df*	*Sig.*	Exp (B)	95% CI	
							Lower	Upper
IOP (median POAG, NTG)	5.256	2.678	3.851	1	0.050	191.756	1.007	36525.673
Age	0.061	0.023	7.424	1	0.006	1.063	1.017	1.111
Age (>40 years/<40 years	−1.161	0.418	7.700	1	0.006	0.313	0.138	0.711
Age (>45 years/<45 years)	−0.924	0.437	4.464	1	0.035	0.397	0.169	0.935
Age (>50 years/<50 years)	−0.981	0.547	3.217	1	0.073	0.375	0.128	1.095
Age (>55 years/<55 years)	0.988	0.809	1.492	1	0.222	2.685	0.550	13.108
Duration of VF performing	−0.229	0.153	2.233	1	0.135	0.795	0.589	1.074
False positive	0.037	0.041	0.836	1	0.360	1.038	0.958	1.124
False negative	−0.024	0.027	0.824	1	0.364	0.976	0.927	1.028
Tested points	0.001	0.005	0.049	1	0.824	1.001	0.992	1.010
Slope 10°	−0.047	0.260	0.033	1	0.856	0.954	0.573	1.587
HOV-zero level	0.116	0.077	2.306	1	0.129	1.123	0.967	1.305
PD	−0.963	0.323	8.909	1	0.003	0.382	0.203	0.718
AD	−0.171	0.309	0.304	1	0.581	0.843	0.460	1.546

**Table 11:** Distribution of affirmative responses to “Symptom Inventory” questionnaire in POAG and NTG patients.

Symptoms Questioned	Answers “Yes” Primary Open-Angle Glaucoma Patients (71)		Answers “Yes” Primary Open-Angle Glaucoma with Normal Intraocular Pressure Patients (39)		Answers “Yes” Total Open-Angle Glaucoma Patients (110)	
	Number	%	Number	%	Number	%
Tearing	34	47.89	15	38.46	49	44.55
Sensation of dry eyes	12	16.90	5	12.82	17	15.45
Sensation of tension in the eye	15	21.13	2	5.13	17	15.45
Scotomas—the lack of a part of the visual field	5	7.04	2	5.13	7	6.36
Limited view: tube/tunnel view	2	2.82	1	2.56	3	2.73
Difficulty in short-distance sight	18	25.35	7	17.95	25	22.73
Difficulty in remote view (to see at a distance)	4	5.63	5	12.82	9	8.18
Disorders in color perception/changes in color intensity	4	5.63	1	2.56	5	4.55
Ebluisare—blindness in bright light	14	19.72	7	17.95	21	19.09
Blindness passing from light to darkness	13	18.31	5	12.82	18	16.36

**Table 12:** Distribution of binomial logistic regression parameters to the symptom questionnaire in OAG (POAG + NTG) and NG patients.

Symptoms Questioned	Parameter Estimate	SE	Wald χ^2^	*df*	*Sig.*	Exp (B)	95% CI	
							Lower	Upper
Tearing	−0.110	0.271	0.165	1	0.685	0.896	0.527	1.523
Sensation of dry eyes	−0.895	0.335	7.143	1	0.008	0.409	0.212	0.788
Sensation of tension in the eye/eye strain	−0.764	0.338	5.097	1	0.024	0.466	0.240	0.904
Scotomas	−0.589	0.496	1.408	1	0.235	0.555	0.210	1.467
Limited view: tube/tunnel view	−0.885	0.704	1.583	1	0.208	0.413	0.104	1.639
Difficulty in short-distance sight	−0.818	0.299	7.470	1	0.006	0.441	0.245	0.793
Difficulty in remote view (to see at a distance)	−1.295	0.412	9.858	1	0.002	0.274	0.122	0.615
Disorders in color perception/in color intensity	−0.499	0.587	0.723	1	0.395	0.607	0.192	1.807
Ebluisare—blindness in bright light	−0.724	0.316	5.228	1	0.022	0.485	0.261	0.953
Ebluisare—blindness passing from light to darkness	−0.992	0.327	9.235	1	0.002	0.371	0.195	0.647

**Table 13:** Distribution of binomial logistic regression parameters to the symptom questionnaire in the POAG and NG patients.

Symptoms Questioned	Parameter Estimate	SE	Wald χ^2^	*df*	*Sig.*	Exp (B)	95% CI	
							Lower	Upper
Tearing	−0.528	0.308	2.947	1	0.086	0.590	0.323	1.078
Sensation of dry eyes	−1.337	0.370	13.019	1	0.000	0.263	0.127	0.543
Sensation of tension in the eye/eye strain	−0.874	0.350	6.223	1	0.013	0.417	0.210	0.829
Scotomas	−0.734	0.541	1.845	1	0.174	0.480	0.166	1.385
Limited view: tube/tunnel view	−1.441	0.780	3.415	1	0.065	0.237	0.051	1.091
Difficulty in short-distance sight	−1.153	0.333	11.988	1	0.001	0.316	0.164	0.606
Difficulty in remote view (to see at a distance)	−2.298	0.551	17.380	1	0.000	0.100	0.034	0.296
Disorders in color perception/in color intensity	−0.130	0.646	0.040	1	0.841	0.878	0.248	3.117
Ebluisare—blindness in bright light	−1.295	0.354	13.364	1	0.000	0.274	0.137	0.548
Ebluisare—blindness passing from light to darkness	−1.459	0.361	16.310	1	0.000	0.232	0.114	0.472

**Table 14:** Distribution of binomial logistic regression parameters to the symptom questionnaire in NTG and NG patients.

Symptoms Questioned	Parameter Estimate	SE	Wald χ^2^	*df*	*Sig.*	Exp (B)	95% CI	
							Lower	Upper
Tearing	−0.361	0.381	0.899	1	0.343	0.697	0.331	1.470
Sensation of dry eyes	−1.113	0.522	4.551	1	0.033	0.329	0.118	0.914
Sensation of tension in the eye/eye strain	−1.982	0.756	6.871	1	0.009	0.138	0.031	0.607
Scotomas	−0.818	0.788	1.077	1	0.299	0.441	0.094	2.067
Limited view: tube/tunnel view	−0.949	1.086	0.764	1	0.382	0.387	0.046	3.252
Difficulty in short-distance sight	−1.114	0.460	5.858	1	0.016	0.328	0.133	0.809
Difficulty in remote view (to see at a distance)	−0.794	0.528	2.263	1	0.132	0.452	0.161	1.272
Disorders in color perception/in color intensity	−1.092	1.078	1.027	1	0.311	0.336	0.041	2.773
Ebluisare—blindness in bright light	−0.799	0.464	2.966	1	0.085	0.450	0.181	1.117
Ebluisare—blindness passing from light to darkness	1.278	0.519	6.056	1	0.014	3.589	1.297	9.930

**Table 15:** Distribution of binomial logistic regression parameters to the symptom questionnaire in the POAG and NTG patients.

Symptoms Questioned	Parameter Estimate	SE	Wald χ^2^	*df*	*Sig.*	Exp (B)	95% CI	
							Lower	Upper
Tearing	−0.385	0.406	0.902	1	0.342	0.680	0.307	1.507
Sensation of dry eyes	−0.324	0.574	0.319	1	0.572	0.723	0.235	2.228
Sensation of tension in the eye/eye strain	−1.600	0.782	4.189	1	0.041	0.202	0.044	0.934
Scotomas	−0.338	0.861	0.154	1	0.695	0.714	0.132	3.861
Limited view: tube/tunnel view	−0.097	1.241	0.006	1	0.938	0.908	0.080	10.343
Difficulty in short-distance sight	−0.440	0.499	0.779	1	0.378	0.644	0.242	1.711
Difficulty in remote view (to see at a distance)	0.901	0.703	1.644	1	0.200	2.463	0.621	9.772
Disorders in color perception	−0.819	1.136	0.520	1	0.471	0.441	0.048	4.088
Ebluisare—blindness in bright light	−0.116	0.513	0.051	1	0.821	0.891	0.326	2.434
Ebluisare—blindness passing from light to darkness	−0.421	0.569	0.549	1	0.459	0.656	0.215	2.001
